# pH Regulator on Digital Microfluidics with Pico-Dosing Technique

**DOI:** 10.3390/bios13110951

**Published:** 2023-10-25

**Authors:** Haoran Li, Tao Peng, Yunlong Zhong, Meiqing Liu, Pui-In Mak, Rui P. Martins, Ping Wang, Yanwei Jia

**Affiliations:** 1 The State Key Laboratory of Analog and Mixed-Signal VLSI, Institute of Microelectronics, University of Macau, Macau 999078, China; yb77457@um.edu.mo (H.L.); liumq@fjirsm.ac.cn (M.L.); pimak@um.edu.mo (P.-I.M.);; 2 Zhuhai UM Science & Technology Research Institute, Zhuhai 519085, China; zumri.taopeng@um.edu.mo; 3 Department of Hepatobiliary Surgery, The First Affiliated Hospital of Guangzhou Medical University, Guangzhou 510120, China; 4 Faculty of Science and Technology–ECE, University of Macau, Macau 999078, China; 5 Instituto Superior Técnico, Unversidade de Lisboa, 1049-001 Lisboa, Portugal; 6 MoE Frontiers Science Center for Precision Oncology, University of Macau, Macau 999078, China

**Keywords:** digital microfluidic, pH regulator, pico-dosing

## Abstract

Real-time pH control on-chip is a crucial factor for cell-based experiments in microfluidics, yet difficult to realize. In this paper, we present a flexible pH regulator on a digital microfluidic (DMF) platform. The pico-dosing technology, which can generate and transfer satellite droplets, is presented to deliver alkali/acid into the sample solution to change the pH value of the sample. An image analysis method based on ImageJ is developed to calculate the delivered volume and an on-chip colorimetric method is proposed to determine the pH value of the sample solution containing the acid-base indicator. The calculated pH values show consistency with the measured ones. Our approach makes the real-time pH control of the on-chip biological experiment more easy to control and flexible.

## 1. Introduction

pH value—the characterization of the hydrogen–ion concentration in a solution—plays an important role in adjusting biological functions, moderating microbial activities, controlling nutrient availability, and regulating chemical behavior [1,2,3,4,5,6]. Close monitoring and precise control of the pH value in a reaction is critical for a reliable and reproducible experimental result. In a laboratory, the pH is normally regulated by titrating acid, alkali, or metal ions directly into a solution or dissolving different amounts of carbon dioxide in the sample. With enough sample volume, the pH value can be digitally monitored with a pH meter or colorimetrically analyzed with a pH indicator.

Nowadays, more and more biochemical analyses are transferred from bench to chip with microfluidic technologies, which can handle minute amounts of precious biochemical samples. Microfluidics has shown its advantages in the biochemical field with its small footprint, low consumption of samples, and fast reaction responses. It has been successfully applied in DNA amplification, chemical synthesis, immunoassays, cell cultures, etc. [7,8,9,10,11,12]. Nevertheless, the transplantation of the traditional pH control and pH detection methods to microfluidic chips faces huge challenges given the small sample volume on-chip, the limited size of the flow channels, and the unique sample manipulation process.

Creative microfluidic techniques have been developed to regulate the pH of a sample drop on-chip, mostly in flow-based channel microfluidics. Welch et al. [13] built microvalves to open or close the flow of the acid and basic solutions for titration. They utilized an extended gate ion-sensitive field-effect transistor and an integrated pseudo-reference electrode as the sensor to monitor pH values. A real-time feedback system was built to turn on or turn off the microvalves for precise pH value control. C. Läritz et al. designed a ${CO}_{2}$-diffusor on-chip, which used the silicon membrane to separate the flow channel and gas chamber while allowing gas diffusion to regulate the pH [14]. However, both methods needed to precisely open and close the fluid or gas channels with microvalves or silicon membranes, significantly increasing the difficulty of chip manufacturing fabrication. 

Instead of direct chemical titrations or gas diffusion, electrolysis could generate the hydrogen and hydroxide ions under a certain voltage to regulate the pH and only required inserting microelectrodes in the flow channel [15]. However, due to the flow of the solution, it was difficult to sustain a stable pH in the solution. In order to obtain a sample with a stable concentration of $H^{+}$ or ${OH}^{-}$, Zhou et al. proposed a droplet-based microfluidic pH regulator [16]. The concentrated electrolyzed ions were encapsulated in droplets for a specific pH value and the pH value of each droplet could be controlled by tuning the pressure pulse and the electrolyzing voltage. Nevertheless, air generated in the electrolysis may affect the subsequent analysis. The solution passing through the electrolysis zone would flow out of the flow channel, resulting in waste of the samples. 

Compared with channel-based microfluidics, digital microfluidics (DMF) can manipulate droplets on an array of electrodes with a convenient external electric control strategy based on electrowetting on dielectric (EWOD) [17,18,19,20,21]. All the samples can be accommodated into droplets for analysis individually on a DMF chip. Various biochemical applications such as DNA amplification, cell culture, immunoassays, and peptide synthesis have been successfully realized in DMF. However, pH detection and regulation have yet to be investigated in DMF. This is because the pH regulation requires adding another solution to the sample. The commonly used method of mixing two solutions in DMF was to merge two droplets, where the volume of the droplet was doubled and the concentration of $H^{+}$ was halved after merging. A specific pH value would require multiple merging and splitting steps, which would undoubtedly increase the complexity of the experimental procedure. Droplets merging in channel-based microfluidics also had the same problem, i.e., inconvenient sample delivery on-chip.

Our recent work developed an on-chip pico-dosing system based on the satellite droplet ejection phenomenon [22]. It realized flexible pL level on-chip sample delivery without affecting the volume of the target droplet. DNA was specifically identified and quantified by the on-chip pico-dosing system. With the adjustable sample delivery volume, the unaltered sample volume and reagents’ concentration, and the convenient electronic control, the pico-dosing system can be a perfect technology for pH regulation on DMF chips. 

**Figure 1:**
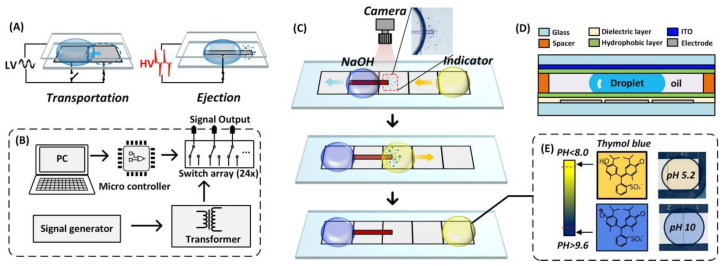
pH regulator system. (**A**) Transportation and ejection of a droplet; (**B**) DMF control system; (**C**) the process of pH regulation; (**D**) the cross-section view of the DMF chip; (**E**) color display of thymol blue.

In this work, we propose a pH regulator utilizing the pico-dosing system on DMF to achieve real-time pH regulation on a chip. Figure 1 illustrates the working principle of the pH regulator.In DMF system, drop manipulation is normally based on EWOD, the wetting effect of a drop on a hydrophobic dielectric surface with an applied electric field of a reasonable strength. When a drop covers two adjacent electrodes where one is activated, the contact angle of the drop on the activated electrode decreases, causing the drop to be out of bal-ance. The drop moves from the inactivated electrode to the activated one to lower its sur-face energy. This means that a drop can be transported, dispensed, mixed, and stored on chip by programming the electrode activation sequence. The transportation of droplets was achieved by a sinusoidal voltage (Figure 1A). When the actuation signal was an alternating current (AC) where the direction of the electric field continuously alternated, the contour of the drop was unstable at high voltage for a defined contact angle saturation (CAS). In addition, when the actuation voltage was above the threshold (a voltage causing CAS), the drop started to vibrate rigorously, and showers of satellite droplets were ejected from the actuated drop. A controlled amount of sodium hydroxide was ejected under a high-voltage pulse actuation signal. The satellite droplet ejection occurred to release the excess energy accumulated in the ac-tuated drop beyond the contact angle saturation, the detailed physical mechanism still needs to be explored, and more detail of the principle and the parametric optimization can be found in our previous work [22].

Figure 1B shows the schematic view of the control system. The ejected satellite droplets were picked up by a sample droplet for the pH titration (Figure 1C). Figure 1D shows the schematic view of the DMF chip. We also developed a pH determination method based on colorimetric analysis by incorporating a pH indicator in the solution (Figure 1E). The real-time pH detection was used for the automatic pH regulation by controlling the amount of hydroxide addition. To the best of our knowledge, this is the first pH control system on DMF. The simple design without the complicated large peripherals such as pumps and valves rendered the system easy to apply. The direct sample addition without affecting the volume made the pH regulation process flexible and convenient, paving the way to an automatic micro–total analysis system. Generally, specific pH values are fundamental to assessing many cellular metabolic pro-cesses, and cellular dysfunction is always associated with abnormal pH values in orga-nelles [23]. The pH fluctuations in the cellular environment may affect normal cell growth [24]. The DMF-based pH regulation method proposed in this study will be essential for cellular metabolism detection and biosensor-based applications.

## 2. Materials and Methods

### 2.1. Materials

Sodium hydroxide (NaOH), thymol blue (TB), and ethanol were purchased from Sigma (St. Louis, MO, USA); 18.2 MΩ deionized (DI) water was purified from tap water with a DI water machine (Milli-Q^®^). 

### 2.2. DMF System Setup

The automated DMF system included self-developed PC software, control electronics, a DMF chip, and a microscope mounted with a camera. An image of the setup can be found in ESI Figure S1. The PC software contained the functions for controlling the switch between different actuation signals, the actuation time, programming for a series of actuations, and other functions for human–computer interactions. The control electronics were on a printed circuit board (PCB). It provided the electric actuation signals, the amplification of the input signal to a desired voltage and waveform, and physical relays for connecting or disconnecting the electric signal to the DMF chip. Two initial actuation signal channels—a sinusoidal wave and a square wave signal—generated by a signal generator (Keysight, Santa Rosa, CA, USA), were amplified by two electromagnetic transformers mounted on the PCB board, which was then connected to the DMF chip for droplet actuation. A field-programmed gate array (FPGA) was employed to transfer data between the control software and the control electronics. The FPGA received the command from the PC software through Bluetooth to turn on or off the physical relays (RS2H-S-DC5) to determine whether to apply the actuation signal to the electrode. The DMF chip mounted on a 3D-printed chip holder was electrically connected with the output port of the control electronics through the flat cable and chip clip. The chip was placed under a microscope (Olympus, Tokyo, Japan) with a camera. The software Cellsens was used for microscope control and image acquisition to observe the droplet manipulation in real-time. 

### 2.3. DMF Chip Fabrication

The pH regulator DMF chip had a sandwiched structure with a top plate made of ITO glass and a bottom plate of glass patterned with an array of chromium electrodes. The droplets were confined in between and immersed in silicon oil (1 cSt). The detailed fabrication process has been described in our previous work [22,25,26] and can be found in ESI, Figure S2. Briefly, patterned electrodes on a glass plate were made by lithography in Shao Guang Chrome Blank (Changsha, China) as the bottom plate. After receiving the bottom plate, a layer of 10 um SU-8 film was coated on top as the dielectric layer. The connecting pad was left as bare electrodes for connecting the DMF chip to the electric control. Then, a patterned SU-8 of 50 um thick was coated on the bottom plate at the corners of each electrode as fences to prevent the droplets from drifting. The alignment was achieved using a mask aligner (ABM, San Jose, CA, USA). Then, a film of Teflon was coated by spin coating 0.5% Teflon AF on top as the hydrophobic layer. Similarly, the ITO glass used as the top plate was also coated with Teflon AF on its conductive surface. Finally, the top and bottom plates were assembled with the hydrophobic sides facing each other. A conductive tape of 200 um was used as the spacer to separate the two plates and form a chamber to accommodate the droplets and the medium oil. 

### 2.4. Preparation of Standard pH Solutions for Colorimetric Analysis

A 0.075% thymol blue indicator solution was prepared by dissolving 0.1 g of thymol blue powder in 20 mL ethanol and diluting it to a final volume of 133 mL with DI water. A stock NaOH solution of 1M was prepared by dissolving 40 mg NaOH in DI water to a final volume of 10 mL. The pH value of the thymol blue solution was measured with a pH meter (Sartorius PB-10, Goettingen, Germany). Then, 0.5 µL of the sample from the solution was loaded onto an electrode on the DMF chip with the same setup in all the experiments in this work for an image capture under a microscope (Olympus, Tokyo, Japan). Then, 1 µL of the 1M NaOH was titrated into a 12 mL indicator solution for another pH value standard solution. Then, 0.5 µL of this titrated solution was loaded onto the chip for another image for colorimetric analysis after the pH meter determined the pH. The process obtained a series of standard pH solutions for colorimetric calibration on-chip. 

## 3. Results

### 3.1. pH Regulator on DMF 

In DMF devices, electrowetting on dielectric (EWOD) is the basic principle to actuate a drop. The strength of the EWOD force can be estimated from the change in the contact angle of a drop on the dielectric surface in the presence of an electric field. Before the contact angle reaches saturation, the Young–Lippmann equation can be used to numerically calculate the contact angle ($\theta_{v}$) as

(1) $$\cos\theta_{v}=\cos\theta_{0}+\frac{1}{\gamma_{LG}}\cdot \frac{\varepsilon_{r}\varepsilon_{0}}{2d}V^{2}$$

where $\theta_{0}$ is the initial contact angle, $\gamma_{LG}$ is the surface tension between liquid and surrounding of the drop, $\varepsilon_{r}$ and $\varepsilon_{0}$ are the permittivity of vacuum and dielectric constant of the dielectric layer, *d* is the thickness of the dielectric layer, and *V* is the applied voltage.

As seen from Equation (1), a higher voltage would generate a larger contact angle difference, therefore a more potent force for drop transportation. For widely used dielectric materials, SU-8 or Parylene C of 8~10 μm thickness, where $\varepsilon_{r}$ is about 3.4. Empirically, a voltage of about 90 V was needed to drive a drop transportation smoothly on a DMF chip with a space height of 200 μm. The contact angle saturation voltage was found to be 160 V. 

For a typical electric signal generator, the upper limit of the output signal was 5 V, which was much lower than demanded and required signal amplification before being applied to a DMF chip. Electromagnetic transformers were utilized for signal amplification with fewer windings on the input side and more windings on the output side. Given the characteristics of an electric magnetic transformer, not only is the voltage raised after transforming, but also the waveform of the electric signal is modified. For a sinusoidal signal, the output was another sinusoidal with a higher voltage amplitude and a phase change of 90°, while, for a square wave input signal, the output signal was a pulse wave with a much higher peak voltage. Table 1 shows the output voltage of different input waveforms. As can be seen, the peak-to-peak voltage of the pulse wave transformed from a square wave is two times higher than that from a sinewave. It has been found that the Young–Lipmann equation only works before the drop reaches the contact angle saturation. When a high AC electric field exceeded the saturation voltage, the contact angle did not change, while showers of tiny droplets would be ejected from the actuated mother drop. This phenomenon was called satellite droplet ejection [27].

**Figure 2:**
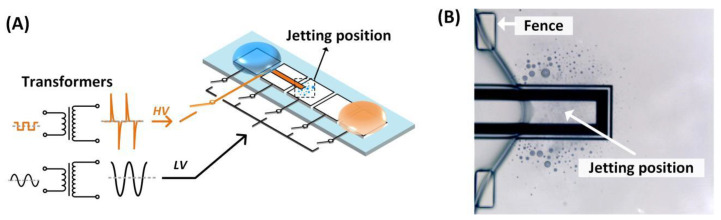
Signal generation and chip design. (**A**) The generation of two kinds of actuation signal and the electric connection of the DMF chip; (**B**) the detail of the jetting position.

To utilize the satellite droplets ejection, we designed two different types of electrodes connecting different waveforms of actuation signals, respectively. As shown in Figure 2A, the conventional square electrodes were arranged to form a path provided with a sinusoidal actuation signal with lower voltage (LV) to actuate the transportation of the droplet. A long bar-shaped electrode (jetting bar) with a width of 100 μm was embedded in the middle of a square electrode. The jetting bar was connected with a peak pulse actuation signal with higher voltage (HV) to eject satellite droplets. It should be noted that the length of the jetting bar exceeded the transportation electrode in which it was embedded so that the ejected droplets were concentrated on one end of the jetting bar (as shown in Figure 2B). The area where the satellite droplets were located was called “jetting position”.

The pH regulator was built based on the satellite droplets ejection phenomenon, as shown in Figure 1. A sandwiched DMF chip was fabricated to accommodate the medium oil, the sample drop, and the medium oil. The transportation or ejection was controlled by the control electronics, which could be programmed to turn on/off the actuation signals individually or simultaneously. The dispensed satellite droplets of acid/alkali were picked up by the target droplet to adjust the pH value. The target drop that picked up the satellite droplets was actuated to move back and forth on several adjacent electrodes to be well-mixed. The pH value was determined by colorimetric analysis. Thymol blue was used as the indicator, which changed color from yellow to blue in the range of pH 8~9.6, as shown in Figure 1E.

### 3.2. pH Determination with Colorimetric Analysis

To determine the pH value of a solution in a particular range with a pH indicator, the color of the solution was compared with the colors of standard solutions at various pH values. However, the color of a drop on microfluidic chips under the microscope was much lighter than the color of the bulk solution off-chip due to the small droplet size. To make the comparison meaningful, we recalibrated the colors of droplets on-chip at different pHs. 

**Figure 3:**
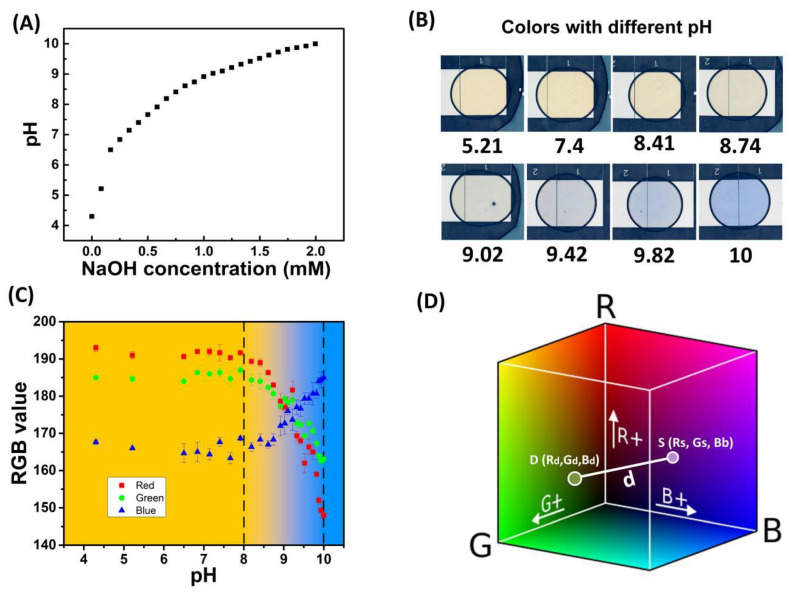
On-chip color calibration of droplets. (**A**) pH value of different NaOH concentrations; (**B**) images of droplets with different pH values; (**C**) standard curve of RGB three-component at different pH values of on-chip droplets; (**D**) RGB color space and spatial distance between two color points. For every NaOH concentration, three droplets were observed and recorded.

Figure 3A shows the pH value measured by a standard pH meter (Sartorius PB-10, Goettingen, Germany) at various concentrations of NaOH mixed with thymol blue, which dissolved in deionized water. It should be noted that the pH value of pure thymol blue solution showed weak acidic characteristics. The pH value reached neutral when the NaOH concentration raised to around 0.2 mM. This uncommon pH distribution might be caused by the lack of buffering capacity of DI water. Due to the deficient ion concentration in deionized water, it is difficult for a pH meter to quickly and accurately measure the pH value. Moreover, CO_2_ dissolves in the solution to form acetic acid, which causes the pH of the solution to decrease. But this acidic phenomenon was unstable and a small amount of titration would return the pH to neutral. As shown in Figure 3A, this abnormal tendency was quickly eliminated with the addition of NaOH. When the NaOH concentrations were above 0.25 mM, the pH values increased gradually with the NaOH. 

The color of drops at each pH value was recorded for colorimetric analysis. Figure 3B shows some typical images at different pHs, but a direct comparison of the color for pH determination was tedious and unprecise. Here, we proposed a novel approach for automatic pH determination based on digital colorimetric analysis. 

The images of drops at various pH values from 4.3 to 10 were analyzed in RGB color, as shown in Figure 3C. As can be seen, when the pH was less than 8, the green (G) and red (R) components of the droplet color were much higher than the blue (B) component, which gave the droplets a yellowish color. As the pH increased to above 8, the red and green components gradually decreased, while the blue component increased until the final solution totally turned blue at pH 10. This calibration result was consistent with the color properties of thymol blue, a yellow-to-blue change in the pH range of 8.0 to 9.6. 

RGB color analysis of the acquired images was used to quantify the color of samples with different pH values. From the relationship between the RGB values and pH values, the functions of $R_{s}(pH)$, $G_{s}\left(pH\right),$ and $B_{s}(pH)$ could be fitted as:

(2) $$R_{s}=-0.16{pH}^{4}+3.53{pH}^{3}-28.6{pH}^{2}+99.15pH+68.24,$$

(3) $$G_{s}=0.04{pH}^{4}-1.86{pH}^{3}+26.6{pH}^{2}-150.8pH+482.67,$$

(4) $$B_{s}=-0.027{pH}^{4}+1.21{pH}^{3}-16.67{pH}^{2}+90.57pH-6.06,$$

where $R_{s}$, $G_{s}$, and $B_{s}$ are the three components of the standard color. 

In the RGB color space, every point represents a color. The distance between two points in the color space represents the similarity of the two colors. As shown in Figure 3D, the distance between the color of a droplet with unknown pH in an experiment and standard color in RGB color space was:

(5) $$d=\sqrt{{\left(R_{d}-R_{s}\right)}^{2}+{\left(G_{d}-G_{s}\right)}^{2}+{\left(B_{d}-B_{s}\right)}^{2})},$$

where *d* is the distance between the standard color and the color of the target droplet with unknown pH; $R_{d}$, $G_{d}$, and $B_{d}$ are the three components of the target droplet color in the RGB color space. Combining Equations (2)–(5), a function of *d* with respect to *pH*, d(*pH*), could be derived, which had an extreme value. The pH value corresponding to the minimum value of d(*pH*) was regarded as the pH value of the unknown sample.

### 3.3. Image Analysis of Satellite Droplets

The pH regulator in this work delivered a certain volume of NaOH into the target drop for pH titration. In our previous work, the delivered volume was obtained from the fluorescence intensity calibration [22]. However, the picking up and calculation method was inapplicable for real-time adjustment. Here, we employed an image analysis method to estimate the total delivered volume by adding up the volume of all the satellite droplets. Free software ImageJ from the National Institutes of Health (NIH) was used for the image analysis. 

**Figure 4:**
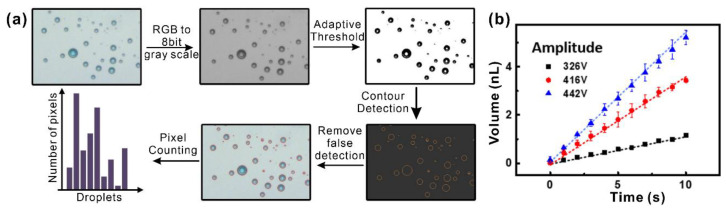
Image analysis for volume determination of satellite droplets. (**a**) The image analysis procedure; (**b**) delivered volume calculated by image analysis under different actuation voltages.

As shown in Figure 4a, the original RGB three-channel image of the satellite droplet was converted into an 8-bit grayscale image. Then the grayscale image was converted into a binary image with only black (0, 0, 0) and white (255, 255, 255) colors. The particle analysis function of ImageJ was used to obtain the size information of each satellite droplet in the image. Through this information, the total volume of satellite droplets was calculated by the formula:

(6) $$V=\Sigma_{\dot{i}}\frac{4}{3}\pi {\left(\sqrt{\frac{S_{i}}{\pi }}\right)}^{3},$$

where $S_{i}$ obtained by ImageJ was the area of the $i^{th}$ droplet provided by ImageJ and *V* was the total volume of the satellite droplets. Here, we regarded the shape of each satellite droplet as an approximate sphere. 

Figure 4b shows the volume delivered using the image analysis method. As can be seen, the delivered volume increased linearly with the ejection time when the other parameters were fixed. For different amplitudes, the sample was delivered in different efficiencies and higher actuation voltage led to more ejection. Please note that the volume calculated with the image analysis may not be consistent with that obtained from the fluorescence calibration. This was due to the fact that, with the image analysis method, some tiny fog-like droplets were neglected by the software, causing a slightly lower volume estimation than the fluorescence calibration method. In addition, a few droplets falling near the gap between the jetting bar and the transportation electrode were also challenging to detect. These deviations are acceptable for basic delivery volume estimation and trend prediction. 

### 3.4. pH Regulation on Chip

**Figure 5:**
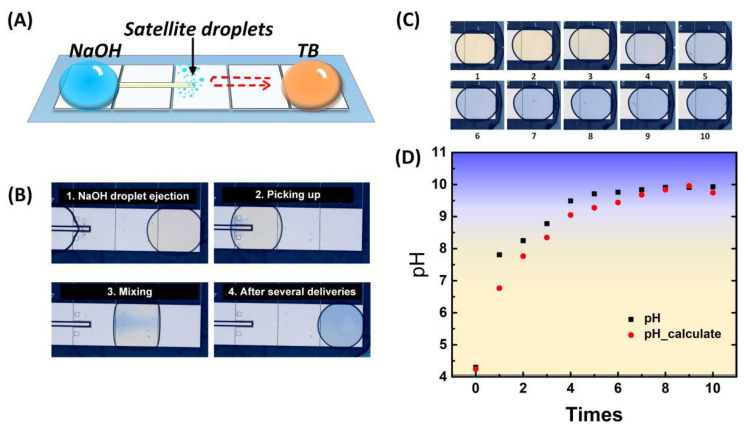
On-chip pH regulation. (**A**) Experimental design; (**B**) operation process of NaOH delivery; (**C**) the color changes with time of target droplet in pH regulation; (**D**) pH value determined by different methods varies with the number of deliveries.

To demonstrate the real-time pH regulation on the DMF chip, 0.5 µL of 1 M NaOH solution and 0.5 µL of thymol blue solution with an initial concentration of 0.075% were loaded on both sides of the ejection bar as the dispensing drop and the target drop, as shown in Figure 5A. 

The NaOH droplet was transported to the jetting position to eject satellite droplets at 440 V for 2 s. The ejected NaOH satellite droplets were picked up by the target droplet containing thymol blue. The target drop added with NaOH moved back and forth on three adjacent transport electrodes to mix until the color was uniform. More deliveries were made for a higher target pH value. The images during the delivery are shown in Figure 5B.

Figure 5C shows the image of the target drop after each delivery. The color gradually changed from yellow to blue with the increased number of deliveries, which reflected the change in the target drop’s pH. To quantify the pH value, we compared the RGB values in the actual images with the standard pH images and determined the pH value at the minimum RGB space distance during the adjustment process. The pH values are shown in Figure 5D. Other than the color analysis, the pH could also be obtained from the added NaOH volume calculated from the image analysis of the ejected satellite droplets. As shown in Figure 5D, the pH values obtained by both methods rose rapidly in the first few deliveries and slowed down in the subsequent deliveries. These results were consistent with the pH titrations, demonstrating the ability of the pH regulator to flexibly adjust the pH of the target drop in real-time. 

In Figure 5D, the calculated values obtained by image analysis were closed with the measured values by colorimetry and showed the same trend. The difference may be caused by the loss of information of some satellite droplets located at the black gap between electrodes during image processing. We anticipate this problem could be solved by optimizing the electrode structure in future work. In addition, further improvement of the image processing algorithm could also improve the accuracy in the calculation of delivery volume, with the hope of regulating the pH value without the pH indicator.

## 4. Conclusions

This paper proposed a new method for pH regulation on DMF chips based on pico-dosing technology. Sodium hydroxide (NaOH) was used as the alkaline regulator to realize the flexible regulation of the pH value of the target droplet containing thymol blue. In practical applications, the pH value of a target drop can be regulated through an external actuation signal for on-chip NaOH titration. On-chip colorimetry was proposed to determine the pH of microscale samples on DMF chips. This method abandoned cumbersome microfluidic chip peripherals, simplified the chip design and on-chip drop operations, and provided a robust approach for optimizing lab-on-a-chip reactions. Testing other ionic solutions, such as HCl, H2SO4, or weak acids for on-chip pH regulation, is still needed in future studies.

## Figures and Tables

**Table 1:** Comparison of output voltage corresponding to the sine and square wave input signals (frequency fixed at 880 Hz).

Input (Vp-p)	1	2	3	4	5	6	7	8	9	10
Output_sine (Vp-p)	36.5	91.9	154.7	216.0	278.0	344.7	413.3	486.7	558.7	634.7
Output_square (Vp-p)	115.3	246.8	371.8	504.0	632.3	751	886.8	1015.0	117.5	1240.0

## Data Availability

The data presented are available on request from the corresponding author.

## Supplementary Materials

The following supporting information can be downloaded at: https://www.mdpi.com/article/10.3390/bios13110951/s1, Figure S1: DMF control platform. (a) System setup. (b) Control electronics on PCB board; Figure S2: Structure and fabrication of DMF chip. (a) The layered structure of DMF chip. (b) Fabrication process of bottom plate.
