# Supplementary material for: pH Regulator on Digital Microfluidics with Pico-Dosing Technique
Source: Biosensors (Basel). 2023 Oct 25;13(11):951. doi: 10.3390/bios13110951 (PMC10669492; doi:10.3390/bios13110951)
Supplement: Supplementary file 1 [file biosensors-13-00951-s001.zip › biosensors-2565095-supplementary.pdf]

biosensors

MDPI

Supplementary Material

pH regulator on digital microfluidics with pico-dosing technique

Haoran Li [a,#], Tao Peng [b,#], Yunlong Zhong [c], Meiqing Liu [a], Pui-In Mak [a,d], Rui P. Martins [a,d,e] , Ping Wang [c,*] and Yanwei Jia [a,d,f,*]

1. System setup

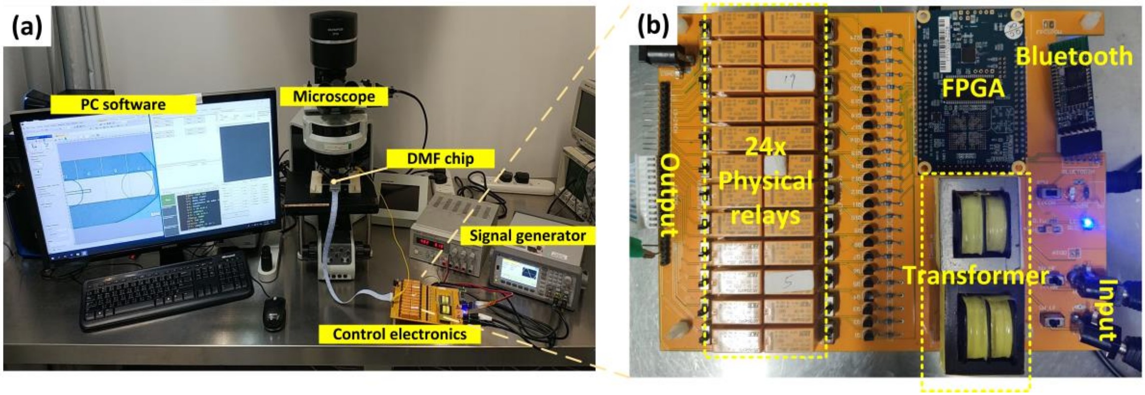

Fig.S1 DMF control platform. (a) System setup. (b) Control electronics on PCB board.

2. Fabrication process

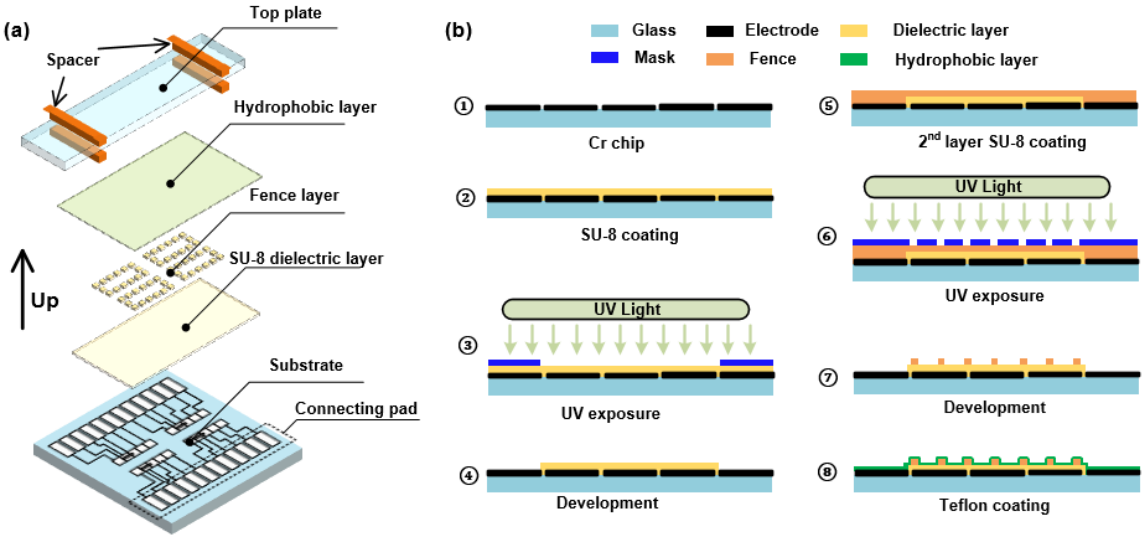

Fig. S2 Structure and fabrication of DMF chip. (a) The layered structure of DMF chip. (b) Fabrication process of bottom plate.
